# Evolution of Genome Architecture in Archaea: Spontaneous Generation of a New Chromosome in *Haloferax volcanii*

**DOI:** 10.1093/molbev/msy075

**Published:** 2018-04-16

**Authors:** Darya Ausiannikava, Laura Mitchell, Hannah Marriott, Victoria Smith, Michelle Hawkins, Kira S Makarova, Eugene V Koonin, Conrad A Nieduszynski, Thorsten Allers

**Affiliations:** 1 School of Life Sciences, University of Nottingham, Queen’s Medical Centre, Nottingham, United Kingdom; 2 National Center for Biotechnology Information, National Library of Medicine, NIH, Bethesda, MD; 3 Sir William Dunn School of Pathology, University of Oxford, Oxford, United Kingdom

**Keywords:** chromosome, genome architecture, multipartite genome, homologous recombination, genome stability, archaea, *Haloferax volcanii*

## Abstract

The common ancestry of archaea and eukaryotes is evident in their genome architecture. All eukaryotic and several archaeal genomes consist of multiple chromosomes, each replicated from multiple origins. Three scenarios have been proposed for the evolution of this genome architecture: 1) mutational diversification of a multi-copy chromosome; 2) capture of a new chromosome by horizontal transfer; 3) acquisition of new origins and splitting into two replication-competent chromosomes. We report an example of the third scenario: the multi-origin chromosome of the archaeon *Haloferax volcanii* has split into two elements via homologous recombination. The newly generated elements are *bona fide* chromosomes, because each bears “chromosomal” replication origins, rRNA loci, and essential genes. The new chromosomes were stable during routine growth but additional genetic manipulation, which involves selective bottlenecks, provoked further rearrangements. To the best of our knowledge, rearrangement of a naturally evolved prokaryotic genome to generate two new chromosomes has not been described previously.

## Introduction

Bacterial genomes usually consist of a single circular chromosome with a unique origin of DNA replication *oriC*, which is recognized by the initiator protein DnaA. Some bacteria, mainly from the phylum Proteobacteria (e.g. *Agrobacterium*, *Brucella*, *Rhizobium*, *Vibrio*), have large secondary replicons termed chromids (Harrison et al. 2010; diCenzo and Finan 2017). Unlike plasmids, chromids are often comparable to the main chromosome in size and carry core genes that are usually found on the main chromosome. However, in contrast to the main chromosome, chromids have been shown to rely exclusively on plasmid-type DNA replication initiation mechanisms (often in the form of a RepABC system), and not on the DnaA/*oriC* system (Egan et al. 2005; Pinto et al. 2012).

Archaea are similar to bacteria in terms of the size and overall organization of their genomes (Koonin and Wolf 2008). However, the core DNA replication proteins found in archaea are more closely related to those of eukaryotes than to their bacterial counterparts. Archaea commonly have more than one origin on the main chromosome and rely on Orc1/Cdc6 replication initiator proteins, which are homologous to the eukaryotic origin recognition complex subunit Orc1 (Makarova and Koonin 2013; Ausiannikava and Allers 2017). Archaeal genomes often have large secondary replicons, which are referred to as mega-plasmids or mini-chromosomes. Unlike bacterial chromids, archaeal mini-chromosomes depend predominantly on Orc1 initiator proteins for their replication, similar to the main chromosome (Ng et al. 1998, 2000; Baliga et al. 2004; Wang et al. 2015).

Eukaryotic genomes consist of multiple chromosomes that are almost always linear and are each replicated from multiple origins. New extrachromosomal elements arise relatively frequently in eukaryotes (Gaubatz 1990; Moller et al. 2015; Turner et al. 2017), but these elements are often transient and low in abundance. Extrachromosomal circular DNAs are common in yeast and may cover up to 23% of the genome (Moller et al. 2015), and cancer cells often generate highly amplified circular mini-chromosomes called double minute chromosomes (Storlazzi et al. 2010).

How did multiple chromosomes with multiple origins evolve? The ancestral state is unlikely to have been a single chromosome with a single origin, but it is the simplest one to consider. (i) If present in multiple copies, a single chromosome could diversify by the accumulation of mutations. (ii) More likely, a new element could be acquired by horizontal transfer—over time, the secondary chromosome would gain core genes from the main chromosome (diCenzo and Finan 2017). (iii) Alternatively, the new element could integrate into the main one, producing a multi-origin chromosome that has the potential to split into two replication-competent chromosomes, thereby giving rise to the state encountered in modern genomes (Egan et al. 2005; diCenzo and Finan 2017). In bacteria, the presence of plasmid-like replication origins on secondary replicons and the uneven distribution of core genes argues against scenario (i) and in favor of scenario (ii) (Harrison et al. 2010). Phylogenetic analysis of the multiple replication origins found on archaeal chromosomes indicates that they were independently acquired through horizontal gene transfer (HGT) and not by duplication of pre-existing origins (Robinson and Bell 2007; Wu et al. 2012), again apparently ruling out scenario (i) and instead supporting scenario (ii). Because features that are common to all eukaryotic replication origins are elusive, little can be deduced about the evolution of eukaryotic genome organization but scenario (iii) might be the most parsimonious.

Whatever the evolutionary scenario, genome architecture is not random in prokaryotes (Rocha 2004, 2008; Press et al. 2016). One of the strongest constraints is the location of replication origins and termination regions; a striking X-shaped pattern of inversions, with endpoints symmetrically located around the origin and terminus of replication, has commonly been observed in bacteria and archaea (Eisen et al. 2000; Novichkov et al. 2009; Repar and Warnecke 2017). It has been shown experimentally that altering the size ratio of the two replication arms (replichores) by >10% is deleterious for *Escherichia coli* (Esnault et al. 2007). A strong bias for codirectionality of transcription and replication, which is thought to reduce the collision of RNA and DNA polymerases, also exists in prokaryotic genomes (Wang et al. 2007; Srivatsan et al. 2010; Ivanova et al. 2015). The distribution of repetitive and mobile elements shapes the genome as well, with both homologous and site-specific recombination acting as a potent driving force of chromosome architecture evolution in bacteria and archaea (Brugger et al. 2004; Papke et al. 2004; Whitaker et al. 2005; White et al. 2008; Bryant et al. 2012; Cossu et al. 2017; Mao and Grogan 2017).

*Haloferax volcanii*, a halophilic archaeon, is a tractable model to study prokaryotic genome plasticity and the evolution of new chromosomes (Mullakhanbhai and Larsen 1975; Charlebois et al. 1991; Hartman et al. 2010). Its main chromosome has three origins, *oriC1*, *oriC2*, and *oriC3* (Norais et al. 2007; Hawkins, Malla, et al. 2013). Three additional origins exist on the three mini-chromosomes, pHV4, pHV3, and pHV1 (Hartman et al. 2010). *Haloferax volcanii* is highly polyploid, with the entire genome present in ∼20 copies (Breuert et al. 2006). Consistent with the highly dynamic nature of archaeal genomes (Redder and Garrett 2006; Bridger et al. 2012), two cases of genome rearrangements have been detected in vivo for *H. volcanii*, namely fusion of the pHV4 mini-chromosome with the main chromosome, and inversion of part of this fused chromosome by recombination between two insertion sequence (IS) elements (Hawkins, Malla, et al. 2013). The former rearrangement has increased the number of replication origins on the main chromosome to four. The involvement of HGT in archaeal genome evolution is evident from the presence of many additional copies of replication genes. In the *H. volcanii* genome, there are 16 *orc* genes encoding the Orc1 initiator protein but only six origins (Hartman et al. 2010; Raymann et al. 2014).

Here we report an unusual genome rearrangement in *H. volcanii*. In our investigation of DNA replication, we generated strains with serial deletions of *orc* genes. It came to our attention that one of these strains had undergone a genome rearrangement. Unexpectedly, the main chromosome split into two parts via homologous recombination between two near-identical *sod* (superoxide dismutase) genes; therefore, it was not due to excision of the integrated pHV4. The two resulting DNA molecules exhibit all the features of *bona fide* chromosomes: they bear replication origins, rRNA loci, and essential core genes.

To the best of our knowledge, the evolution of a new chromosome without interspecies HGT has so far not been observed in prokaryotes. Thus, we have witnessed in vivo a realization of the scenario (iii) posited above: a multi-origin chromosome splits into two replication-competent chromosomes. This finding contrasts with our previous report showing fusion of the pHV4 mini-chromosome with the main chromosome (Hawkins, Malla, et al. 2013) and demonstrates that genome rearrangements do not inexorably lead to larger chromosomes. Instead, they can give rise to the multi-origin/multi-chromosome state encountered in modern genomes.

## Results

### Large-Scale Genome Rearrangement in the Strain Deleted for Orc1/Cdc6 Initiator Gene *orc5*

**Fig. 1.**
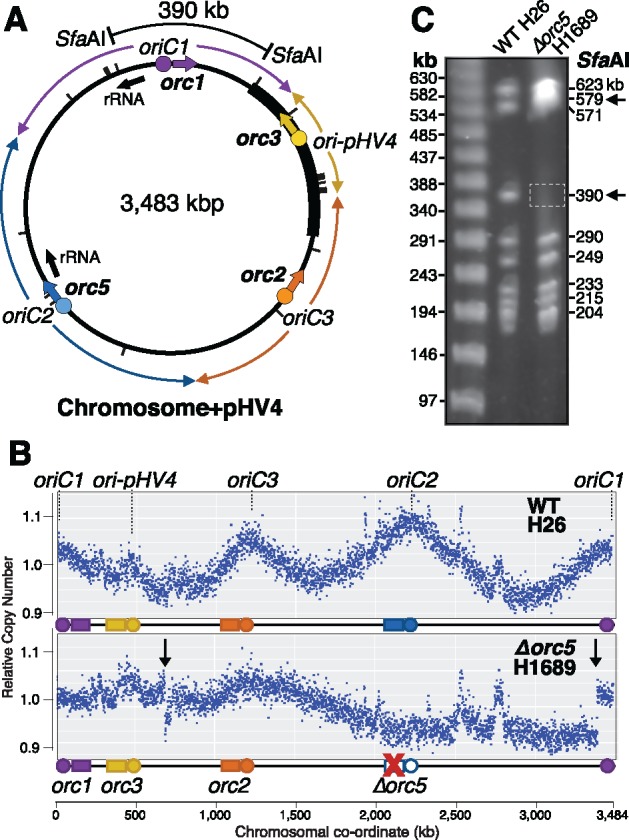
Genome rearrangement of *Δorc5* strain. (*A*) Location of replication origins and adjacent *orc* genes on *Haloferax volcanii* main chromosome (+pHV4). Positions of the two rRNA loci are indicated with black arrows. The integrated pHV4 mini-chromosome is indicated by a thick line. The eight replichores representing the direction of replication forks are shown by colored arrows, corresponding to their respective origins. *Sfa*AI sites are indicated by tick marks. (*B*) Replication profiles of the *Δorc5* mutant H1689 and a reference wild-type (WT) laboratory strain H26. The number of reads is plotted against the chromosomal location. The linearized *H. volcanii* chromosome showing positions of *oriC* and *orc genes* is shown below (colored as in *A*). Two discontinuities in the *Δorc5* replication profile are indicated by vertical arrows. (*C*) Restriction fragment length polymorphisms in WT and *Δorc5* strain as shown by digestion with *Sfa*AI and PFGE. The 390 kb *Sfa*AI fragment (shown on the map in panel *A*) is absent from the digest of *Δorc5* DNA, and a novel 579 kb *Sfa*AI fragment is present; these bands are indicated by arrows.

In our study of Orc1-type initiator proteins and their role in DNA replication in *H. volcanii*, we focused on the four *orc* genes, *orc1*, *orc5*, *orc2*, and *orc3*, which are genetically linked to the four chromosomal origins, *oriC1*, *oriC2*, *oriC3*, and *ori-pHV4*, respectively (fig. 1*A*). The four origins create eight replichores on the chromosome, with *oriC1* being the most active origin and *ori-pHV4* the least (Hawkins, Malla, et al. 2013). We obtained replication profiles by marker frequency analysis using whole genome sequencing (Muller et al. 2014). We noted that upon deletion of *orc5* gene, which is located next to *oriC2*, the mutant strain H1689 had acquired large-scale genome rearrangements. This was manifested as two clear discontinuities in the replication profile (indicated by arrows in fig. 1*B*;Skovgaard et al. 2011), when compared with the wild type (WT).

To verify the genome rearrangement by an independent method, we performed restriction digests with *Sfa*AI and analyzed the fragment sizes by pulsed field gel electrophoresis (PFGE). We have previously used this method to detect genome rearrangements in *H. volcanii* (Hawkins, Malla, et al. 2013). We observed the disappearance of a band corresponding to a 390 kb fragment, and the appearance of a novel 579 kb fragment in the *Sfa*AI digest of *Δorc5* DNA, confirming a large-scale genome rearrangement (fig. 1*C*).

### New Genome Architecture of *Δorc5* Strain

**Fig. 2.**
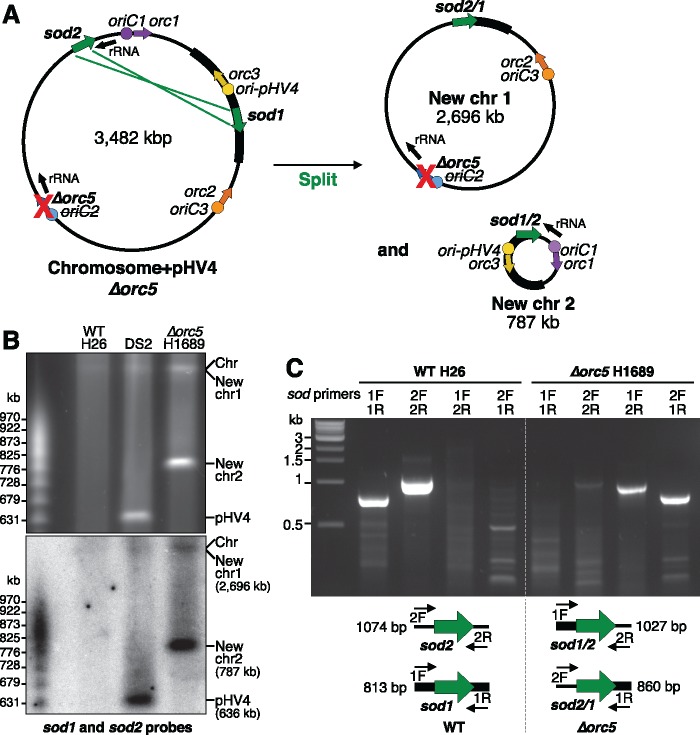
Novel genome architecture of *Δorc5* strain. (*A*) Scheme for outcome of recombination between *sod1* and *sod2* genes to split the main chromosome (+pHV4) and generate two new chromosomes (new chr 1 and new chr 2). (*B*) PFGE and Southern blot confirming two new chromosomes in *Δorc5* strain. Intact genomic DNA of wild isolate DS2, WT H26 and *Δorc5* H1689 strains was probed with *sod1* and *sod2* sequences. (*C*) Recombination of *sod1* and *sod2* genes in Δ*orc5* strain H1689 was confirmed by end-point PCR using primers to unique sequences flanking *sod1* and *sod*. The identity of the PCR products was validated by DNA sequencing.

The two interruptions in the replication profile of *Δorc5* mutant (fig. 1*B*) correspond to the locations of the *sod1* (HVO_A0475; 689201–689803 bp) and *sod2* genes (HVO_2913; 3385084–3385683 bp). The *sod1* and *sod2* superoxide dismutase genes are 603 bp and 600 bp, respectively, and have 100% nucleotide sequence identity (apart from the initial 8 bp); however, their flanking sequences are unique. This provides an opportunity for intrachromosomal homologous recombination of the *sod1* and *sod2* genes, and two outcomes are possible: splitting of the main chromosome into two circular replicons (termed new chr 1 and new chr 2, fig. 2*A*), or chromosomal inversion of the region between the two *sod* genes. Given that the two *sod* genes are in the same orientation (direct repeats), only the former outcome is possible, as the latter would require the *sod* genes to be arranged as inverted repeats.

To investigate the genome architecture of the *Δorc5* strain, intact genomic DNA was analyzed by PFGE and a Southern blot was probed with *sod1* and *sod2* sequences (fig. 2*B*). In the wild isolate DS2 (Mullakhanbhai and Larsen 1975), the *sod1* and *sod2* genes are located on pHV4 and the main chromosome, respectively. In the WT laboratory strain H26, pHV4 is fused with the main chromosome and therefore both *sod* genes are on the same molecule (Hawkins, Malla, et al. 2013). In DNA prepared from the *Δorc5* strain H1689, the *sod1* and *sod2* probes hybridized with two molecules that correspond in size to new chr 1 (2,696 kb) and new chr 2 (787 kb). Using PCR with primers to the unique sequences flanking *sod1* and *sod2*, we determined that these two genes underwent recombination in the Δ*orc5* strain (fig. 2*C*). DNA sequencing of the PCR products confirmed that the unique flanking sequences of *sod1* and *sod2* had been exchanged in the *Δorc5* strain.

**Fig. 3.**
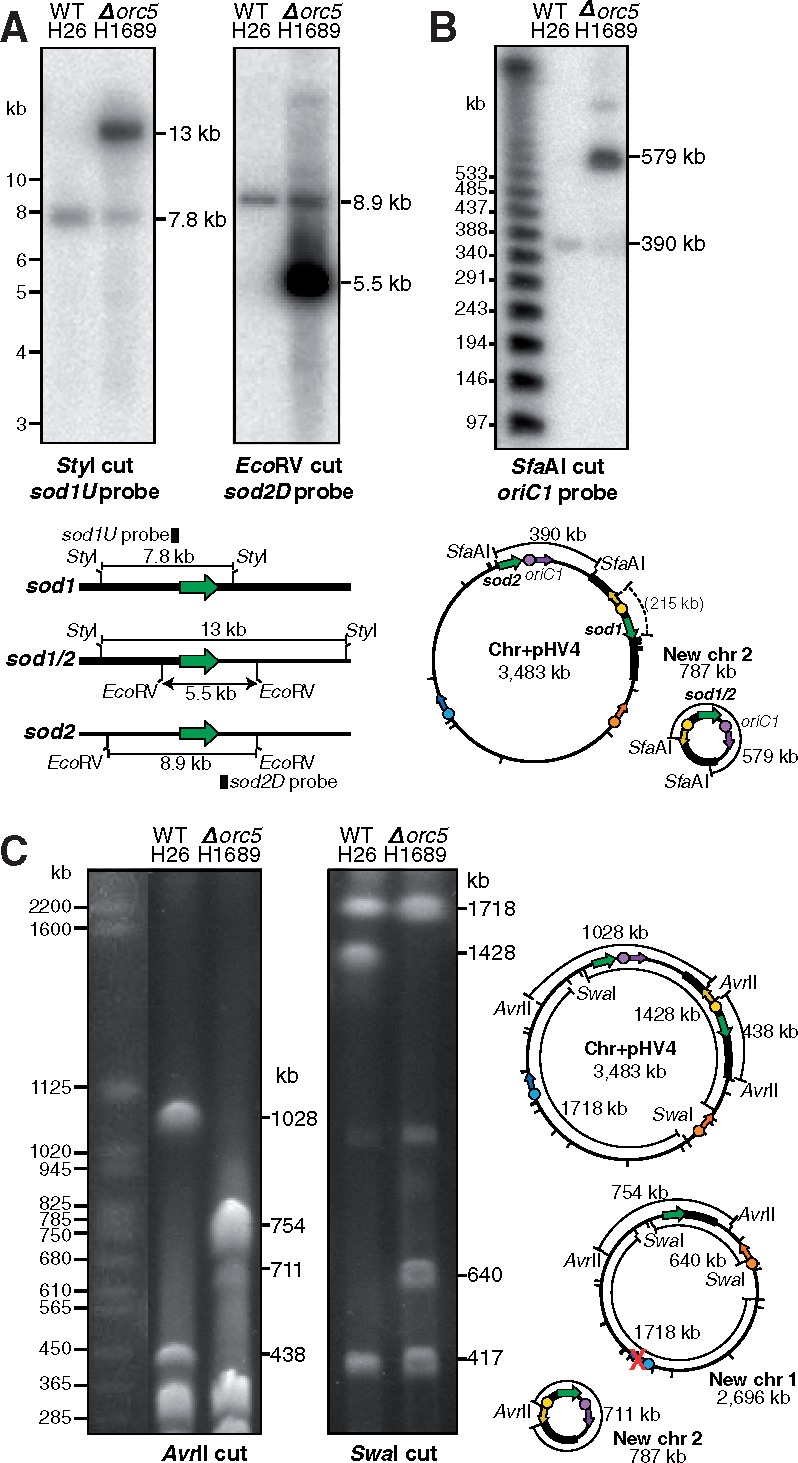
Genome architecture of the *Δorc5* strain is polymorphic. (*A*) Southern blot conforming location of breakpoints of genome rearrangement in Δ*orc5* strain. Genomic DNA of WT H26 and *Δorc5* H1689 was digested with *Sty*I or *Eco*RV and probed with sequences adjacent to *sod1* or *sod2*, respectively. A WT-sized band is present in the *Δorc5* lanes. (*B*) Southern blot of PFGE confirming relocation of *oriC1* to new chr 2 in Δ*orc5* strain. *Sfa*AI-digested DNA of WT H26 and *Δorc5* H1689 strains was probed with sequences adjacent to *oriC1*. Relevant *Sfa*AI sites are indicated on the maps, the new chr 1 does not hybridize with *oriC1* (map not shown). A faint 390 kb WT-sized band is present in the *Δorc5* lane. (*C*) PFGE confirming new genome architecture of *Δorc5* strain. Genomic DNA of WT H26 and *Δorc5* H1689 was digested with *Avr*II or *Swa*I. Relevant *Avr*II and *Swa*I sites are indicated on the outside and inside of chromosome maps, respectively. The 417 bp *Swa*I fragment is found on pHV3 (not shown), which is not affected by the genome rearrangement.

We constructed maps of the rearranged chromosomes (new chr 1 and new chr 2) and analyzed the predicted *sod1/sod2* break points in the *Δorc5* mutant by restriction digests and Southern blotting. As expected, a *Sty*I digest generated one band of 7.8 kb in the WT and a larger 13 kb fragment (plus a faint WT-sized band) in the *Δorc5* strain, which hybridize with a probe adjacent to *sod1* (fig. 3*A*). Similarly, an *Eco*RV digest of DNA from the WT strain generated a fragment of 8.9 kb, which hybridizes with a probe adjacent to *sod2* gene, whereas a smaller 5.5 kb fragment (plus a faint WT-sized band) was seen in the *Δorc5* strain (fig. 3*A*). The presence of the faint fragment of WT size in both digests of the *Δorc5* mutant suggests that the genome architecture of this strain is not monomorphic, and that the two states (with and without genome rearrangement), coexist in the population.

To confirm the splitting of the chromosome into two circular replicons, genomic DNA was digested with *Sfa*AI, analyzed by PFGE and a Southern blot was probed with the *oriC1* downstream region (fig. 3*B*). In the WT, this probe will hybridize with a fragment of 390 kb that includes *sod2*. If the main chromosome is split into two, the 390 kb fragment will be fused with a 215 kb fragment that includes *sod1*, to generate a product of 579 kb. Such a rearrangement would account for the disappearance of the 390 kb band, and the appearance of a novel 579 kb band, as seen in the *Sfa*AI digest in figure 1*C*. The *Sfa*AI-digested *Δorc5* DNA in figure 3*B* showed the presence of such a 579 kb band that hybridizes with the *oriC1* probe. A faint 390 kb fragment corresponding to the WT was also present in the *Δorc5* sample, indicating that the genome architecture of this strain is not monomorphic, confirming the observation made in figure 3*A*.

To further confirm fragmentation of the chromosome into two replicons, genomic DNA was digested with *Avr*II and *Swa*I, and the fragments were analyzed by PFGE (fig. 3*C*). The two largest *Avr*II fragments of WT are 1,028 kb and 438 kb, and include the *sod2* and *sod1* genes, respectively. When the main chromosome is split into two elements, the largest fragments are 754 kb and 711 kb, and are found on new chr 1 and new chr 2, respectively. The *Avr*II digest of *Δorc5* DNA generated two such fragments of 711 kb and 754 kb, alongside the disappearance of fragments of 1,028 kb and 438 kb. The largest *Swa*I fragments of WT are 1,718 kb, 1,428 kb, and 417 kb (the latter is found on pHV3, which is not affected by the genome rearrangement). Splitting the main chromosome into two would eliminate the 1,428 kb *Swa*I fragment and generate a new fragment of 640 kb on new chr 1; these fragments were observed in the *Swa*I digest of *Δorc5* DNA.

Taken together, the PCR and restriction digests indicate that ectopic recombination between the two *sod* genes has led to fragmentation of the main chromosome into two circular replicons. However, the genome architecture of the *Δorc5* strain is polymorphic; that is, a WT chromosome is still present alongside the two new elements.

### *orc5* Deletion Does Not Increase Rate of Large-Scale Genome Rearrangements

**Fig. 4.**
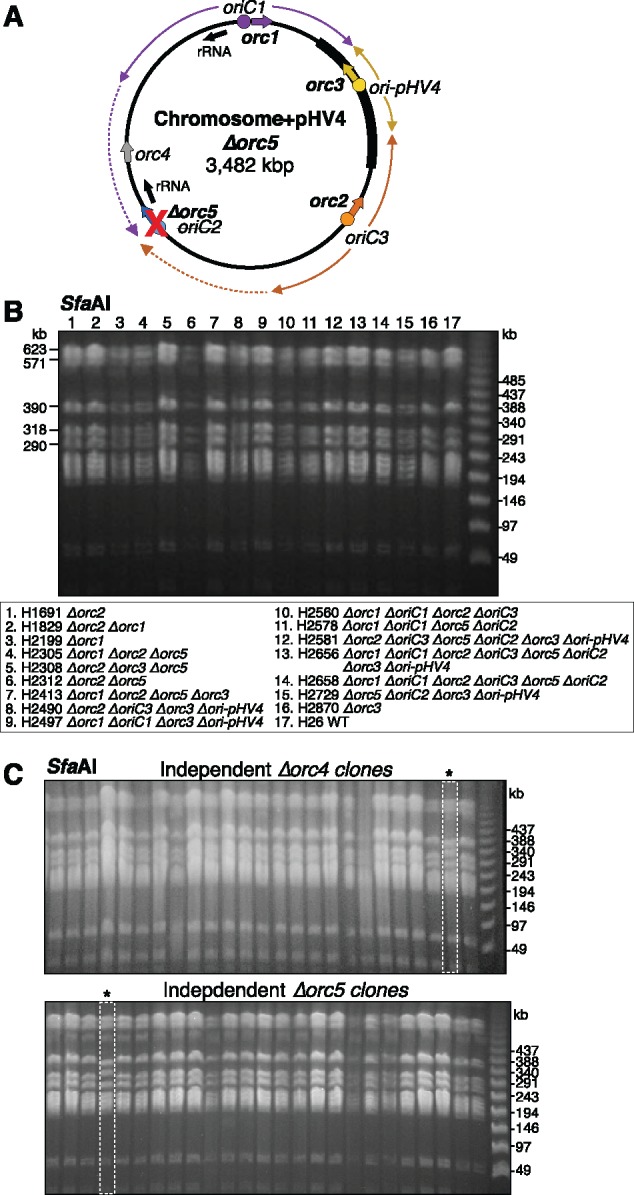
Deletion of *orc5* does not increase the rate of genome rearrangement. (*A*) Scheme showing new replichores in the absence of *orc5* (replichores and rRNA loci indicated as in fig. 1*A*). (*B*) *Sfa*AI restriction fragment length polymorphisms were not seen in unrelated strains with different combinations of *orc* and *oriC* deletion. Strain genotypes are indicated below. (*C*) *Sfa*AI-digested genomic DNA of 25 independently derived *Δorc4* mutants and 25 independently derived *Δorc5* mutants. Representative images, the *Δorc4* clone and *Δorc5* clone with a genome rearrangement are indicated by an asterisk.

The genome rearrangement in the *Δorc5* strain might have been provoked by asymmetric and unbalanced replichores. In the archaeon *Sulfolobus islandicus*, deletion of *orc1-1* or *orc1-3* genes abolishes replication initiation from the adjacent *oriC1* or *oriC2* origins, respectively (Samson et al. 2013). A functional linkage of *orc* genes and origins is also found in *H. volcanii*: the replication profile in figure 1*B* shows that deletion of *orc5* abolishes replication initiation from *oriC2*, which is adjacent to *orc5*. The replichores that derive from the remaining origins *oriC1*, *oriC3* and *ori-pHV4* are predicted to be highly asymmetrical and unbalanced (fig. 1*A* vs. fig. 4*A*). Furthermore, in an *Δorc5* strain, transcription of the rRNA locus that is located adjacent to *oriC2* might no longer proceed in the same direction as DNA replication, provoking head-on collisions of the transcription and replication machinery. Thus, the absence of *orc5* might make the genome unstable and prone to rearrangements. However, the *Δorc5* strain H1689 shows no major growth defects. The growth rate was determined by competition assay to be 5.5% slower than the WT strain (data not shown). This decrease in growth rate is comparable to the 4% growth defect previously reported for a *ΔoriC2* strain, which does not have a genome rearrangement (Hawkins, Malla, et al. 2013).

To test the effect of asymmetric (unbalanced) replichores, we investigated the scale of genome rearrangements in strains with different combinations of *orc* and origin deletions. A total of 16 additional strains were analyzed by *Sfa*AI digestion and PFGE. In all 16 strains, the five largest bands generated by *Sfa*AI were identical in the size to those seen in the WT strain (fig. 4*B*). Therefore, only the *Δorc5* strain underwent a large-scale genome rearrangement. This rearrangement could have occurred by chance or due to the deletion of *orc5*, which potentially might increase the rearrangement rate.

This hypothesis was tested statistically. As an initial control, we estimated the rate of spontaneous genome rearrangement during *H. volcanii* genome manipulation, by testing 100 independent mutants where the *orc4* gene had been deleted. This gene was chosen because it is not expected to play a role in DNA replication: it is not located next to a replication origin or actively transcribed genes, and as judged by synonymous codon usage (SCU), was acquired by HGT (Hartman et al. 2010). Only 1 of the 100 *Δorc4* clones tested exhibited large-scale genome rearrangements as determined by *Sfa*AI digestion (fig. 4*C*). The same analysis was conducted with 115 independently generated *Δorc5* mutants, and only one of the 115 clones tested exhibited a genome rearrangement (fig. 4*C*). When combined with the *Δorc5* strain H1689, the estimated rate of large-scale genome rearrangements in the absence of *orc5* is 1.7% (2/116), which is not statistically different from the 1% background rate obtained with *Δorc4* deletion (*P*-value 0.65, chi-squared test). Thus, deletion of *orc5* and any associated change in the size of the replichores does not appear to lead to an increase in large scale genome rearrangements.

### Evolution of New Chromosomal Architecture in *Δorc5*-Derivative Strains

**Fig. 5.**
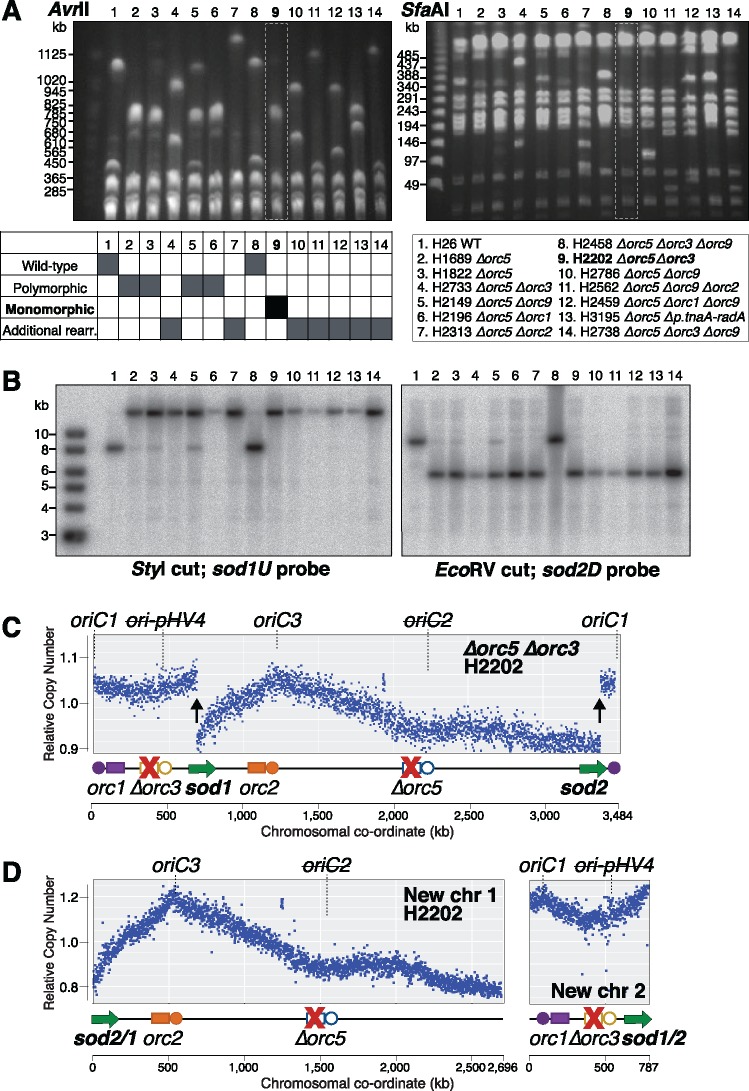
New genome architectures of *Δorc5* derivatives. (*A*) *Avr*II and *Sfa*AI digests of genomic DNA from derivatives of *Δorc5* strain H1689 identifying four different genome states. Strain genotypes and genome architecture state is indicated below, polymorphic and monomorphic refer to strains with H1689-type genome rearrangements. The monomorphic *Δorc5 Δorc3* strain H2202 is indicated. (*B*) Southern blots showing that additional genome rearrangements in derivatives of *Δorc5* strain H1689 did not involve recombination of the *sod* gene region. Genomic DNA was digested with *Sty*I or *Eco*RV and probed with sequences adjacent to *sod1* or *sod2*, respectively (for key to restriction fragments, see fig. 3*A*). (*C*) Replication profile of *Δorc5 Δorc3* strain H2202 (lane 9 in panels *A* and *B*) where the genome is in a monomorphic state. Labeled as in figure 1*B*, the two discontinuities in the replication profile are indicated by vertical arrows. (*D*) Replication profile of *Δorc5 Δorc3* strain H2202 remapped to sequences corresponding to new chr 1 and new chr 2.

In our study of Orc1-type initiator proteins, we generated many strains that were derived from the *Δorc5* mutant H1689. As we show here, H1689 has a large-scale genome rearrangement but its chromosomal architecture is polymorphic, whereby the two new elements co-exist with the parental chromosome. The genetic manipulation of *H. volcanii* includes selective bottlenecks and extensive propagation (Bitan-Banin et al. 2003; Allers et al. 2004), giving an opportunity for polymorphic genome states to be resolved, and potentially for further large-scale rearrangements to occur. Indeed, DNA digests with *Avr*II and *Sfa*AI showed that strains derived from the *Δorc5* mutant H1689 exhibit notable genome dynamics. We observed fragments corresponding to the WT chromosome, fragments similar to those observed in the *Δorc5* strain H1689, as well as fragments of new sizes (fig. 5*A*). To determine whether these new genome fragments had arisen by further recombination between the *sod* genes, we carried out a Southern blot of this region (fig. 5*B*).

A total of four states were observed in the *Δorc5* derivatives. 1) In seven strains (lanes 4, 7, 10, 11, 12, 13, 14), additional genome rearrangements were detected by *Avr*II and *Sfa*AI restriction digests (fig. 5*A*), but these rearrangements did not involve the *sod* gene region (fig. 5*B*). 2) Three strains (fig. 5*B*, lanes 3, 5, 6) had preserved the polymorphic genome architecture of the *Δorc5* strain H1689 (lane 2). 3) In one strain (lane 8), the genome architecture reverted to the original WT state (lane 1). 4) In another strain (lane 9), the new chromosomal elements that appeared in the *Δorc5* strain were now present in a monomorphic state. We obtained the replication profile of this monomorphic strain H2202 (*Δorc5 Δorc3*, lane 9). Two clear discontinuities were observed in the same location as those seen previously with the (polymorphic) *Δorc5* strain H1689 (compare fig. 5*C* vs. fig. 1*B*).

The replication profile of the *Δorc5 Δorc3* strain H2202 was remapped to sequences corresponding to new chr 1 and new chr 2 (fig. 5*D*). There is a clear peak at *oriC3* in the profile of new chr 1, which is deleted for *orc5* (adjacent to *oriC2*) but retains *orc2* (adjacent to *oriC3*). Similarly, there is a clear peak at *oriC1* in the profile of new chr 2, which is deleted for *orc3* (adjacent to *ori-pHV4*) but retains *orc1* (adjacent to *oriC1*).

### Newly Generated Genome Elements Have Features of Bona Fide Chromosomes

To date, six genome elements have been described in *H. volcanii* (table 1). The original strain DS2 contains the main chromosome, pHV4, pHV3, pHV2, and pHV1 (Charlebois et al. 1991). The laboratory strain features a new element that was generated by fusion of the main chromosome with pHV4 (Hawkins, Malla, et al. 2013). Here, we describe the generation of two new replicons, which result from the fission of the fused main/pHV4 chromosome. This genome rearrangement results from ectopic recombination between the near-identical *sod* genes and not due to excision of the integrated pHV4. Do the new replicons qualify as mega-plasmids, chromids, or mini-chromosomes?

**Table 1.** Distribution of Features on Genome Elements in *H. volcanii* Wild Isolate DS2, Laboratory Strain H26, and Δorc5 Strain H1689.

Strain(s)	Genome Element	Size, bp	Number of Genes	SCU, Rare Codons	GC Content	LACA Genes	rRNA Loci	Replication Origins
DS2	Chromosome	2,847,757	2,960	7.3%	66.6%	37.3%	2	*oriC1*, *oriC2*, *oriC3*
DS2	pHV4	635,786	636	15.5%	61.7%	28.3%	0	*ori-pHV4*
H26	Chromosome + pHV4	3,482,975	3,596	8.7%	65.7%	35.5%	2	*oriC1*, *oriC2*, *oriC3*, *ori-pHV4*
H1689	New chr1	2,695,880	2,781	8.3%	66.1%	37.4%	1	*oriC2*, *oriC3*
H1689	New chr2	787,095	815	10.3%	64.6%	33%	1	*oriC1*, *ori-pHV4*
DS2, H26, H1689	pHV3	437,906	380	7.7%	65.5%	35.9%	0	*ori-pHV3*
DS2, H26, H1689	pHV1	85,092	88	26.3%	55.5%	18%	0	*ori-pHV1*

Note.—New genomic elements generated by fission of the fused chromosome + pHV4 are designated as New chr1 and New chr2. The fraction of rare codons was calculated from SCU tables for each genome element (Hartman et al. 2010). The fraction of LACA genes was calculated with cut-off probability of 0.75 (Wolf et al. 2012).

In prokaryotic genomes, chromosomal status is based on the presence of essential and conserved genes, as well as size, copy number, replication control, and evolutionary history (Egan et al. 2005; Harrison et al. 2010). We analyzed the distribution of these features on the new genome elements. As a measure of evolutionary history, we used SCU (Hartman et al. 2010). Local variations in SCU can result from mutation and selection, but a pronounced bias is usually due to HGT from another species as indicated by a large fraction of rare codons. As a measure of gene conservation, we calculated the fraction of genes on each new chromosome that have been mapped back to the genome of the last archaeal common ancestor (LACA; Wolf et al. 2012).

Table 1 indicates that splitting of the fused chromosome generated two replicons that are broadly similar in terms of SCU and the fraction of LACA genes. Both replicons retain an rRNA locus as well as multiple DNA replication origins and *orc* genes. The smaller element retains essential DNA replication genes coding for MCM (HVO_0220), both subunits of polymerase D (HVO_0003, HVO_0065), the large subunit of primase (HVO_0173), PCNA (HVO_0175), and two out of the three subunits of the RFC clamp loader (HVO_0145, HVO_0203); the larger element contains genes coding for polymerase B (HVO_0858), GINS (HVO_2698), the small subunit of primase (HVO_2697), and the histone gene (HVO_0520). Thus, both new genome elements comply with the definition of a chromosome (diCenzo and Finan 2017).

## Discussion

The first DNA replication origin to be identified in archaea was described in 2000 for *Pyrococcus abyssi* (Myllykallio et al. 2000). At the time, it was proposed that archaea and bacteria share a “standard” prokaryotic genome architecture, comprising a single circular chromosome with a unique origin of replication (Vas and Leatherwood 2000). However, this view was overly simplistic. It has since become clear that archaeal genomes can consist of multiple chromosomes, each with single or multiple origins (Ausiannikava and Allers 2017). This is perhaps best exemplified by the genome architecture of *H. volcanii*, which has one large chromosome with three origins and three mini-chromosomes with one origin each (table 1). About 10% of bacteria have more than one replicon (diCenzo and Finan 2017), the best studied example being *Vibrio cholerae* which has a large chromosome and a smaller chromid, each with one origin (Jha et al. 2012). In both *H. volcanii* and *V. cholerae*, genome rearrangements have been documented where two replicons have fused to become one. We have previously reported that during generation of the *H. volcanii* laboratory strain, the pHV4 mini-chromosome fused with the main chromosome by recombination (Hawkins, Malla, et al. 2013). In *V. cholerae*, fusion of the chromosome with the chromid can be induced deliberately or can occur spontaneously. Such spontaneous fusions arise as suppressors of mutations that affect DNA replication (Val et al. 2014), but naturally occurring *V. cholerae* strains with a single chromosome have also been reported (Xie et al. 2017).

Here we describe a genome rearrangement in *H. volcanii* that led to the generation of a new chromosome. The main chromosome, which in the laboratory strain includes the integrated pHV4 mini-chromosome, has split into two parts. The two resulting DNA molecules exhibit all the features of *bona fide* chromosomes: they bear DNA replication origins, rRNA loci, and essential core genes. The genome rearrangement that gave rise to the new chromosome was not a simple reversal of the integration of pHV4, which had occurred by recombination between two identical ISH18 ISs (Hawkins, Malla, et al. 2013). Instead, the genome rearrangement reported here occurred via homologous recombination between the near-identical *sod1* and *sod2* genes. In the wild isolate DS2, these two genes are located on pHV4 and the main chromosome, respectively, but in the laboratory strain they are located on the same DNA molecule.

Phylogenetic analysis of bacterial genomes indicates that additional chromosomal elements arise relatively rarely but once a viable state is achieved, they remain stable over long evolutionary intervals (Harrison et al. 2010; diCenzo and Finan 2017). It is unclear how the stability of the genome is maintained in the multipartite state. Genetic engineering experiments in bacteria have shown that when parts of a multipartite genome are fused, growth rates remain largely unaffected (Guo et al. 2003; Val et al. 2012). This finding is consistent with our observation on the absence of a major growth defect in any of the strains described above. However, multipartite genomes have the potential to be highly dynamic because homologous genes are often found on different (or the same) chromosomal elements, providing ample opportunity for recombination.

The constraints on genome architecture, such as the need to coordinate DNA replication with transcription, might be a reason for the observed stability of multipartite genomes. The fission or fusion of genome elements can potentially cause unbalanced replichores (which will be exacerbated by the relocation of replication termination zones), conflicts between replication and transcription, and/or changes in gene dosage. In archaea such as *H. volcanii*, the equidistant location of replication origins on the chromosome could reflect the evolutionary advantage in maintaining such a spatial arrangement. Surprisingly, we observed no immediate effect on genome stability in *H. volcanii* when the replichores are unbalanced. The genome stability was assessed in strains with different combinations of *orc* deletions, and there was no measurable change in the rate of genome rearrangement following deletion of *orc5*. This finding contrasts with bacterial systems, where replichore imbalance has been shown to lead to genome instability and reduced fitness (Esnault et al. 2007; Dimude et al. 2016). For example, an *E. coli* strain where the origin was moved to an ectopic site has been found to harbor a large chromosomal inversion (Ivanova et al. 2015).

Several reasons might account for the lack of deleterious effects of replichore imbalance in *H. volcanii*. 1) In contrast to bacteria, which have discrete *Ter* replication termination sites, archaea and eukaryotes have broad termination zones where converging replication forks meet (Duggin et al. 2011). This is most likely a consequence of having multiple origins per chromosome, and allows for greater flexibility in replication initiation. 2) Apart from the highly transcribed rRNA genes, transcription in *H. volcanii* is not consistently co-orientated with replication (Hartman et al. 2010). Such an arrangement is both more important and easier to maintain in bacteria, which have a single origin per chromosome. 3) The polyploid nature of *H. volcanii* genome (where each chromosome is present in 15–20 copies) could also account for the lack of genome instability, because deleterious genome rearrangements can be restored by gene conversion with a WT copy of the affected chromosome. 4) Little is known about the regulation of replication initiation in archaea. *Haloferax volcanii* might use some origins as a “backup” to compensate for replichore imbalance, thereby avoiding any potential conflicts. Alternatively, differential origin usage within one cell, where some chromosomes use one origin and others use a different one, would ameliorate unbalanced replichores. Both scenarios—compensatory and stochastic origin firing—have been observed in eukaryotic replication (Hawkins, Retkute, et al. 2013). 5) Recombination-dependent replication, which is used in the absence of origins, leads to dispersed initiation throughout the genome and may relieve the spatial constraints on replication origins. Thus, replichore imbalance would have only minor effects on the viability of *H. volcanii*.

Nonetheless, it is notable that the Δ*orc5*-derivative strains exhibited considerable genome plasticity and the ability to evolve to different chromosome architectures (fig. 5). The two new chromosomes were stable during routine growth but new rounds of genetic manipulation appeared to provoke further rearrangements. Following transformation, a selectable marker will initially be present on only one of the 20 chromosome copies. This selectable marker will then spread throughout the genome by gene conversion, and may carry with it genetically linked rearrangements. Therefore, the selective bottleneck of genetic manipulation might allow a new chromosome architecture to become monomorphic.

Eukaryotic cells contain multiple linear chromosomes that are replicated from multiple origins. For this type of genome architecture to arise, three steps are required (but not necessarily in this order): multiplication of origins, multiplication of chromosomes, and linearization of chromosomes. Given the shared evolutionary history of eukaryotes and archaea, it is not surprising that two of these three features are found in archaeal genomes as well. Up to four replication origins can be present on some archaeal chromosomes, and multiple chromosomes that use an Orc-type replication initiation mechanism co-exist in haloarchaeal species; however, no archaeon with linear chromosomes has been found to date. Here, we show that an increase in the number of circular chromosomes is easily achievable through natural evolution. To the best of our knowledge, rearrangement of a naturally evolved prokaryotic genome that generates two new chromosomes, each with pre-existing multiple origins that depend on the same type of replication initiation, has not been described previously. Interestingly, the *H. volcanii* genome might already contain an imprint of a similar event, where the ancestral chromosome fragmented leading to the generation of a new chromosome. Indeed, the pHV3 mini-chromosome has one Orc-dependent replication origin, a native SCU and GC content similar to the main chromosome, and a high proportion of LACA genes (table 1); thus, the generation of pHV3 is compatible with the recombinational route described here.

Newly generated chromosomal elements must find effective solutions for segregation and replication, and the ability to spread throughout a population would be beneficial. Haloarchaea have developed potential solutions to these challenges. The proclivity of *H. volcanii* to use recombination-dependent replication in the absence of origins weakens the requirement for newly generated chromosomal elements to maintain balanced replichores, or even origins (Hawkins, Malla, et al. 2013). *Haloferax volcanii* does not strictly depend on orderly segregation of its chromosomes, because its genome is highly polyploid and new chromosomal elements can rely on random partitioning into daughter cells; furthermore, archaea lack the centromeres found on eukaryotic chromosomes. Haloarchaea have a remarkable capacity for rapid genome evolution by HGT. The exchange of up to 530 kb of DNA between different *Haloferax* species has been detected after cell fusion (Naor et al. 2012), thus providing the opportunity for a newly generated chromosome (and eventually, a new species) to arise. And because archaeal origins are nearly always linked to an *orc* gene encoding their cognate initiator protein, a “foreign” chromosome will be efficiently replicated in its new host cell. The remarkable plasticity of haloarchaeal genomes thus presents a test bed for probing the evolution of genome organization and replication initiation.

## Materials and Methods

### Strains and Plasmids

*Haloferax volcanii* strains (table 2) were grown at 45 °C on complete (Hv-YPC) or casamino acids (Hv-Ca) agar, or in Hv-YPC broth, as described previously (Allers et al. 2004). Isolation of genomic and plasmid DNA, and transformation of *H. volcanii*, were carried out as described previously (Allers et al. 2004). Standard molecular techniques were used (Sambrook and Russell 2001). Deletion mutants were constructed and confirmed by colony hybridization and/or Southern blotting as described previously (Allers et al. 2004). Plasmids for gene deletion are shown in table 3 and were generated by PCR using oligonucleotides shown in table 4. Probes for Southern blots are shown in table 5. Growth competition assays were carried out as described previously (Hawkins, Malla, et al. 2013).

**Table 2.** *H. volcanii* Strains.

Strain	Genotype	Derivation	Use
DS2		(Mullakhanbhai and Larsen 1975)	Wild isolate
H26	*ΔpyrE2*	(Allers et al. 2004)	Standard laboratory strain
H53	*ΔpyrE2 ΔtrpA*	(Allers et al. 2004)	Laboratory strain, *trpA* deletion
*Strains with large-scale genome rearrangements*			
H1689	*ΔpyrE2 Δorc5*	H26 pTA1375	Deletion of *orc5*, large-scale genome rearrangement
H1822	*ΔpyrE2 Δorc5 ΔtrpA*	H1689 pTA95	*trpA* deletion in *Δorc5* strain
H2149	*ΔpyrE2 Δorc5 Δorc9*	H1689 pTA1433	*orc9* deletion in *Δorc5* strain
H2196	*ΔpyrE2 Δorc5 Δorc1*	H1689 pTA1610	*orc1* deletion in *Δorc5* strain
H2202	*ΔpyrE2 Δorc5 Δorc3*	H1689 pTA1373	*orc3* deletion in *Δorc5* strain
H2313	*ΔpyrE2 Δorc5 ΔtrpA Δorc2:: trpA+*	H1822 pTA1632	*orc2* deletion in *Δorc5* strain
H2458	*ΔpyrE2 Δorc5 Δorc3 Δorc9*	H2202 pTA1433	*orc9* deletion in *Δorc5 Δorc3* strain
H2459	*ΔpyrE2 Δorc5 Δorc1 Δorc9*	H2196 pTA1433	*orc9* deletion in *Δorc5 Δorc1* strain
H2562	*ΔpyrE2 Δorc5 Δorc9 Δorc2*	H2149 pTA1379	*orc2* deletion in *Δorc5 Δorc9* strain
H2733	*ΔpyrE2 Δorc5 Δorc3 ΔtrpA*	H2202 pTA95	*trpA* deletion in *Δorc5 Δorc3* strain
H2738	*ΔpyrE2 Δorc5 Δorc3 Δorc9 ΔtrpA*	H2458 pTA95	*trpA* deletion in *Δorc5 Δorc3 Δorc9* strain
H2786	*ΔpyrE2 Δorc5 Δorc9 ΔtrpA*	H2149 pTA95	*trpA* deletion in *Δorc5 Δorc9* strain
H3195	*ΔpyrE2 Δorc5 p.tnaA-radA+*	H1689 pTA1837	Tryptophan-inducible *radA* allele in *Δorc5* strain
*Strains with wild-type genome architecture*			
H1691	*ΔpyrE2 Δorc2*	H26 pTA1379	Deletion of *orc2*
H1829	*ΔpyrE2 Δorc4:: trpA+*	H53 pTA1452	Deletion of *orc4*
H2197	*ΔpyrE2 Δorc1 Δorc2*	H2199 pTA1610	*orc2* deletion in *Δorc1* strain
H2199	*ΔpyrE2 Δorc1*	H26 pTA1610	Deletion of *orc1*
H2203	*ΔpyrE2 Δorc2 Δorc3*	H1691 pTA1373	*orc3* deletion in *Δorc2* strain
H2304	*ΔpyrE2 Δorc3 Δori-pHV4*	H26 pTA1631	Deletion of *ori-pHV4* and *orc3*
H2305	*ΔpyrE2 Δorc1 Δorc2 Δorc5*	H2197 pTA1375	*orc5* deletion in *Δorc1 Δorc2* strain
H2308	*ΔpyrE2 Δorc2 Δorc3 Δorc5*	H2203 pTA1375	*orc5* deletion in *Δorc2 Δorc3* strain
H2312	*ΔpyrE2 Δorc2 Δorc5*	H1691 pTA1375	*orc5* deletion in *Δorc2* strain
H2413	*ΔpyrE2 Δorc1 Δorc2 Δorc5 Δorc3*	H2305 pTA1373	*orc3* deletion in *Δorc1 Δorc2 Δorc5* strain
H2490	*ΔpyrE2 Δorc3 Δori-pHV4 Δorc2 oriC3*	H2304 pTA1692	*oriC3* and *orc2* deletion in *Δori-pHV4 Δorc3* strain
H2492	*ΔpyrE2 Δorc2 ΔoriC3*	H26 pTA1692	Deletion of *oriC3* and *orc2*
H2494	*ΔpyrE2 Δorc1 ΔoriC1*	H26 pTA1691	Deletion of *oriC1* and *orc1*
H2497	*ΔpyrE2 Δorc3 Δori-pHV4 Δorc1 ΔoriC1*	H2304 pTA1691	*oriC1* and *orc1* deletion in *Δori-pHV4 Δorc3* strain
H2560	*ΔpyrE2 Δorc2 ΔoriC3 Δorc1 ΔoriC1*	H2492 pTA1691	*oriC1* and *orc1* deletion in *ΔoriC3 Δorc2* strain
H2561	*ΔpyrE2 Δorc2 ΔoriC3 Δorc3 Δori-pHV4 Δorc1 ΔoriC1*	H2490 pTA1691	*oriC1* and *orc1* deletion in *ΔoriC3 Δorc2 Δori-pHV4 Δorc3* strain
H2578	*ΔpyrE2 Δorc1 ΔoriC1 Δorc5 ΔoriC2*	H2494 pTA1712	*oriC2* and *orc5* deletion in *ΔoriC1 Δorc1* strain
H2579	*ΔpyrE2 Δorc5 ΔoriC2*	H26 pTA1712	Deletion of *oriC2* and *orc5*
H2581	*ΔpyrE2 Δorc2 ΔoriC3 Δorc3 Δori-pHV4 Δorc5 ΔoriC2*	H2490 pTA1712	*oriC2* and *orc5* deletion in *ΔoriC3 Δorc2 Δori-pHV4 Δorc3* strain
H2656	*ΔpyrE2 Δorc1 ΔoriC1 Δorc2 ΔoriC3 Δorc3 Δori-pHV4 Δorc5 ΔoriC2*	H2561 pTA1712	*oriC2* and *orc5* deletion in *ΔoriC1 Δorc1 ΔoriC3 Δorc2 Δori-pHV4 Δorc3* strain
H2658	*ΔpyrE2 Δorc1 ΔoriC1 Δorc2 ΔoriC3 Δorc5 ΔoriC2*	H2560 pTA1712	*oriC2* and *orc5* deletion in *ΔoriC1 Δorc1 ΔoriC3 Δorc2* strain
H2729	*ΔpyrE2 Δorc3 Δori-pHV4 Δorc5 ΔoriC2*	H2579 pTA1631	*ori-pHV4* and *orc3* deletion in *ΔoriC2 Δorc5* strain
H2870	*ΔpyrE2 Δorc3*	H26 pTA1373	Deletion of *orc3*
H3380	*ΔpyrE2 ΔtrpA Δorc5:: trpA+*	H53 pTA1633	Deletion of *orc5*

**Table 3.** Plasmids.

Plasmid	Relevant Properties	Derivation
pTA95	Integrative plasmid for *trpA* gene deletion	(Allers et al. 2004)
pTA131	Integrative plasmid based on pBluescript II, with *pyrE2^+^* marker	(Allers et al. 2004)
pTA298	pUC19 with *trpA^+^* marker flanked by *Bam*HI sites	(Lestini et al. 2010)
pTA333	pUC19 with *Sac*I-*Nsp*I chromosomal fragment containing *orc4* gene	This study
pTA415	pBluescript II SK+ with *Mlu*I chromosomal fragment containing *hel308* helicase gene	This study
pTA416	pBluescript II with *Sac*I chromosomal fragment containing *orc5* and *oriC2*	(Norais et al. 2007)
pTA419	pTA131 with *Nhe*I-*Eco*RI fragment of pTA416 containing *orc5* and *oriC2*	This study
pTA1100	pBluescript II with *Aci*I chromosomal fragment containing *orc2* and *oriC3*	(Hawkins, Malla, et al. 2013)
pTA1329	pTA131 with *Δori-pHV4* construct	(Hawkins, Malla, et al. 2013)
pTA1343	pTA131 with *p.tnaA-radA^+^:: hdrB^+^* construct flanked by upstream and downstream *radA* regions	(Hawkins, Malla, et al. 2013)
pTA1370	pBluescript II SK+ with *Hind*III-*Kpn*I chromosomal fragment containing *orc1* gene and *oriC1* origin	This study
pTA1371	pBluescript II SK+ with *BstB*I chromosomal fragment containing *orc3* gene	This study
pTA1373	pTA131 with *Δorc3* construct, comprising *Cla*I*-Bam*HI fragment of upstream flanking region of *orc3* and *Bam*HI*-Xba*I fragment of downstream flanking region of *orc3*, PCR amplified from pTA1371	This study
pTA1375	pTA131 with *Δorc5* construct, comprising *Kpn*I-*Bam*HI fragment of downstream flanking region of *orc5* and *Bam*HI-*Xba*I fragment of upstream flanking region of *orc5*, PCR amplified from pTA416	This study
pTA1379	pTA131 with *Δorc2* construct, comprising *Kpn*I*-Bam*HI upstream flanking region of *orc2* and *Bam*HI*-Xba*I fragment of downstream flanking region of *orc2*, PCR amplified from pTA1100	This study
pTA1431	pTA131 with inactivation of unique *Bam*HI site in MCS by filling-in with Klenow	This study
pTA1432	pBluescript II SK+ with *Not*I chromosomal fragment containing *orc9* gene	This study
pTA1433	pTA1431 with *Δorc9* construct, comprising *Xba*I*-Bst*XI upstream flanking region of *orc9* and *Xba*I*-Bst*XI fragment of downstream flanking region of *orc9*, PCR amplified from pTA1432	This study
pTA1610	pTA131 with *Δorc1* construct, comprising *Kpn*I*-Bam*HI upstream flanking region of *orc1* and *Bam*HI*-Xho*I fragment of downstream flanking region of *orc1*, PCR amplified from pTA1370	This study
pTA1631	*Δorc3 Δori-pHV4* construct, where *orc3* upstream region of pTA1373 was replaced by *Kpn*I*-Bam*HI fragment of *ori-pHV4* upstream region from pTA1329	This study
pTA1632	pTA1379 with insertion of *Bam*HI *trpA^+^* fragment from pTA298	This study
pTA1633	pTA1375 with insertion of *Bam*HI *trpA^+^* fragment from pTA298	This study
pTA1691	pTA131 with *Δorc1 ΔoriC1* construct, comprising *Stu*I*-Bam*HI upstream flanking region of *oriC1* and *Bam*HI*-Xba*I fragment of downstream flanking region of *orc1*, PCR amplified from pTA1370	This study
pTA1692	pTA131 with *Δorc2 ΔoriC3* construct, comprising *Aat*II*-Bam*HI upstream flanking region of *oriC3* and *Bam*HI*-Kpn*I fragment of downstream flanking region of *orc2*, PCR amplified from pTA1100	This study
pTA1712	pTA131 with *Δorc5 ΔoriC2* construct, comprising *Xba*I*-Bam*HI upstream flanking region of *oriC2* and *Bam*HI*-Xba*I fragment of downstream flanking region of *orc5*, PCR amplified from pTA416	This study
pTA1837	pTA131 with *p.tnaA-radA^+^* construct. *Xba*I-*Bam*HI fragment of *hdrB^+^* marker was removed from pTA1343, and 890 bp *Eco*RV*-Pvu*II fragment of *radA* upstream flanking region (PCR amplified from H26 genomic DNA) was used to replace 315 bp *Eco*RV*-Pvu*II fragment of *radA* upstream flanking region in pTA1343	This study
pID19T-HVO_2042	pTA131 with *Δorc4:: trpA^+^* construct, comprising *Xho*I-*Hind*III fragment of upstream flanking region of *orc4* and *Bam*HI-*Xba*I fragment of downstream flanking region of *orc4*, PCR amplified from H26 genomic DNA, joined using *Hind*III-*Bam*HI *trpA^+^*fragment	Jerry Eichler

**Table 4.** Oligonucleotides.

Primer	Sequence (5′–3′)	Relevant Properties	Use
MHorc3F1	CGTTCAtCGATTTGACGAGGTCATCCACG	*orc3* deletion, upstream	pTA1373
MHorc3R1	GTCCCGGaTCCCGATAGATCTCGGTGTCC	*orc3* deletion, upstream	pTA1373
MHorc3F2	ACGACTggATCcAGCAGTAGGTAGGTCG	*orc3* deletion, downstream	pTA1373
MHorc3R2	CCTCCGtCtAGAACACGACGTGCGCGACC	*orc3* deletion, downstream	pTA1373
MHorc2F1	CAGCGgTAcCGACCCGTCGCAGAGGTACG	*orc2* deletion, upstream	pTA1379
MHorc2R1	CGCAGGatCCGAGGCCGCCTGACCCCACG	*orc2* deletion, upstream	pTA1379
MHorc2F2	GCTCGgAtCCGGCGCATTAGCGTCGGTCC	*orc2* deletion, downstream	pTA1379, pTA1692
MHorc2R2	CCGAGGTctAGACATTTCGAGGGGCGG	*orc2* deletion, downstream	pTA1379, pTA1692
MHorc5F1	GTGCTAGGTacCTGAACACCCATAAGTG	*orc5/oriC2orc5* deletions, downstream	pTA1375, pTA1712
MHorc5R1	GCTCGAGGATCCGGACGTGGTGAGGGACG	*orc5/oriC2orc5* deletions, downstream	pTA1375, pTA1712
MHorc5F2	GTGAAGAGGaTCcTCGCTGGCGTTAGGC	*orc5* deletion, upstream	pTA1375
MHorc5R2	GGGGAAtcTAGAGAACCGGAAAACCCGG	*orc5* deletion, upstream	pTA1375
delorc9USR	TCTTCGGGaTCCTCCCTCATCGAG	*orc9* deletion, upstream	pTA1433
delorc9DSF	CGGTCGgAtCCGCGCCATCTCGCTCG	*orc9* deletion, downstream	pTA1433
pBSR3	ACCCCAGGCTTTACACTTTATGC	*orc9* deletion, downstream	pTA1433
pBSF2	TTAAGTTGGGTAACGCCAGGG	*orc9* deletion, upstream, and *oriC1orc1* deletion, downstream	pTA1433, pTA1691
MHorc1F1	ACGAGCgGTaCCGGACGATGCGCGCCGGC	*orc1* deletion, downstream	pTA1610
dorc1DF	AGAACGggaTCCCGAAGTCCGACGC	*orc1/oriC1orc1* deletion, downstream	pTA1610, pTA1691
MHorc1F2	GTTCCCGGaTCCCCTCGTGCGCCGCCTCG	*orc1* deletion, upstream	pTA1610
MHorc1R2	CCACAGTCTaGaCCTCGCCGCAGTAGCCG	*orc1* deletion, upstream	pTA1610
oriC1-BamHL	GTACTCCGGATCCATGCTCGGTATCCG	*oriC1orc1* deletion, upstream	pTA1691
pBSR2	CGCGCAATTAACCCTCACTAAAG	*oriC1orc1* and *oriC3orc2* deletions, upstream	pTA1691, pTA1692
oriC3-BamHL	GGTGTCGGAtCcCGGCTTTCGCGTTCCG	*oriC3orc2* deletion, upstream	pTA1692
OriC2-BamL	CCGGTCTCGGATCCAACTTAGCTCTCACTCG	*oriC2orc5* deletion, upstream	pTA1712
OriC2-XbaR	CGACCCTCTAGAGCGAGGCGAGGTCGCCCC	*oriC2orc5* deletion, upstream	pTA1712
5′HVO_2042_XhoI_F	cccctcgagTCTTTGCAGTCTATTTCCTTC	*orc4* deletion, upstream	pID19T-HVO_2042
5′HVO_2042_HindIII_R	gggaagcttACGTGTTGCAGACCTGTATAC	*orc4* deletion, upstream	pID19T-HVO_2042
3′HVO_2042_BamHI_F	cccggatccCCCACAGAACAGATGAAGTG	*orc4* deletion, downstream	pID19T-HVO_2042
3′HVO_2042_XbaI_R	gggtctagaCGTGCTTCCGAGTCAGAAAC	*orc4* deletion, downstream	pID19T-HVO_2042
radAUSNdeR	TTCTGCCATAtgCAGTCGTTCCGCCTATACCC	*p.tnaA: radA+* construct, upstream	pTA1837
radAextraUS	AGACCAGCTGAGTTCCGATGGGGCTGTTC	*p.tnaA: radA+* construct, upstream	pTA1837
sod1F	AGTACAGGCCGAACTCGACGACGCC	*sod1* Southern blot probe, diagnostic PCR and sequencing of *sod1*	Figure 2*B*, *C*
sod1R	TCTCACGGTAACCTGTGGTCGCGCG	*sod1* Southern blot probe, diagnostic PCR and sequencing of *sod1*	Figure 2*B*, *C*
sod2F	GAAATCGCCGACGCCGTCTCGACG	*sod2* Southern blot probe, diagnostic PCR and sequencing of *sod2*	Figure 2*B*, *C*
sod2R	GAGCAGTTTCGGACCTTCGTCGGCG	*sod2* Southern blot probe, diagnostic PCR and sequencing of *sod2*	Figure 2*B*, *C*
sod1 US-left	ACAGGCTCCGAACGTATCAT	*sod1U* Southern blot probe	Figures 3*A*, 5*B*
sod1 US-right	CAGTCGGTGAGTCCCTGTAA	*sod1U* Southern blot probe	Figures 3*A*, 5*B*
sod2 DS-left	GATGACCTCCGCGACCTC	*sod2D* Southern blot probe	Figures 3*A*, 5*B*
sod2 DS-right	GGGTCGCTGAACAGGTCC	*sod2D* Southern blot probe	Figures 3*A*, 5*B*

**Table 5.** Probes.

Probe	Usage	Location	Source
*sod1*	Figure 2*B*	*sod1* gene	813 bp PCR using sod1F and sod1R
*sod2*	Figure 2*B*	*sod2* gene	1074 bp PCR using sod2F and sod2R
*sod1U*	Figures 3*A*, 5*B*	Upstream of *sod1* gene	359 bp PCR using sod1 US-left and sod1 US-right
*sod2D*	Figures 3A, 5*B*	Downstream of *sod2* gene	347 bp PCR using sod2 DS-left and sod2 DS-right
*oriC1*	Figure 3*B*	Downstream of *oriC1* origin	763 bp *Sty*I fragment of pTA415
*orc4*	Confirmation of *orc4* deletion by colony hybridization	*orc4* gene	959 bp *Bgl*II-*Pst*I fragment of pTA333
*orc5*	Confirmation of *orc5* deletion by colony hybridization	*orc5* gene	784 bp *Aat*II fragment of pTA419

### Screening for Genome Rearrangements in *Δorc5* and *Δorc4*-Deleted Backgrounds

Twelve independent “pop-in” strains were generated using *Δorc5* and *Δorc4* plasmids pTA1375 and pID19T-HVO_2042, respectively, and ten deletion (“pop-out”) strains were derived from each “pop-in.” Gene deletions were confirmed by colony hybridization with the relevant *orc5* or *orc4* probes. The deletion strains were assessed for *Sfa*AI restriction fragment length polymorphisms by PFGE.

### Marker Frequency Analysis by Deep Sequencing

For exponential-phase samples, strains were grown overnight in Hv-YPC broth, diluted 500-fold in fresh media and incubated at 45 °C with vigorous aeration until an A650 of 0.4, then diluted 500-fold in fresh media and grown until an A650 of 0.2. For a stationary-phase sample, a WT culture was grown at 45 °C for 3 days until saturation (no further increase in A650). Genomic DNA was isolated from 50 ml cultures followed by phenol: chloroform extraction as described previously (Hawkins, Malla, et al. 2013). Marker frequency analysis was performed by Deep Seq (University of Nottingham) using Illumina HiSeq 2000 sequencing to measure sequence copy number. Enrichment of uniquely mapping sequence tags was calculated (in 1-kb windows) for exponentially growing samples relative to a stationary phase WT sample, to correct for differences in read depth across the genome (Skovgaard et al. 2011; Muller et al. 2014). Sequence reads were mapped to the *H. volcanii* genome and replication profiles were calculated as described previously (Hawkins, Malla, et al. 2013).

### Pulsed Field Gel Electrophoresis

For PFGE, genomic DNA was prepared in agarose plugs and digested as described previously (Hawkins, Malla, et al. 2013). For analysis of intact genomic DNA, agarose plugs were subjected to 100 Gy of γ radiation using a ^137^Cs source (Gammacell 1000), to linearize circular chromosomes (Beverley 1989). PFGE was performed using a CHEF Mapper apparatus (Bio-Rad). Intact and *Sfa*AI-digested DNA fragments were separated on a 1.2% agarose gel in 0.5× TBE at 14 °C, with a gradient voltage of 6 V/cm, linear ramping, an included angle of 120°, initial and final switch times of 0.64 s and 1 min 13.22 s, respectively, and a run time of 40 h (intact DNA) or 20 h 46 min (*Sfa*AI-digested DNA). *Avr*II-digested and *Swa*I-digested genomic DNA were separated on 1% agarose gel in 0.5× TBE at 14 °C, with a gradient voltage of 6 V/cm, linear ramping, an included angle of 120°, initial and final switch times of 1 min and 2 min, respectively, and a run time of 24 h. The gel was stained with ethidium bromide.
